# Cancer in Youth Living With HIV (YLWHIV): A Narrative Review of the Access to Oncological Services Among YLWHIV and the Role of Economic Strengthening in Child Health

**DOI:** 10.3389/fpubh.2020.00409

**Published:** 2020-08-14

**Authors:** Ruth G. N. Katumba, Ozge Sensoy Bahar, Kimberly J. Johnson, Fred M. Ssewamala

**Affiliations:** ^1^School of Medicine, Washington University in St. Louis, St. Louis, MO, United States; ^2^Brown School, Washington University in St. Louis, St. Louis, MO, United States

**Keywords:** HIV/AIDS, malignancies, youth, sub-Saharan Africa, access to cancer services, economic strengthening

## Abstract

Youth Living with HIV/AIDS (YLWHIV) have a higher risk of developing immunodeficiency related illnesses including certain cancers than their general population counterparts of the same age. This narrative review of current available literature describes factors associated with pediatric access to oncological services, and the role economic strengthening could play in improving health outcomes for this vulnerable population. Findings suggest that both HIV-infected and -uninfected children living in low and middle-income countries struggle with access and adherence to cancer treatment and care. Cost of treatment is a major barrier to access and adherence. Asset-building savings programs may increase financial security and subsequently result in better health outcomes although they have not been utilized to improve access to cancer treatment.

## Introduction

The HIV/AIDS pandemic continues to pose a significant threat to global public health (1). According to the World Health Organization (WHO), ~37.9 million people were living with HIV at the end of 2018, with 1.7 million new infections within that year globally (2). Furthermore, 940,000 people died from HIV-related causes globally in 2017 (2). Africa is the most affected region, with more than two thirds of the total new HIV infections and people living with HIV worldwide (2, 3).

Youth (0–24 years) are greatly affected by the HIV pandemic. This can be explained by their diverse risk factors whereby children can acquire HIV from their infected mothers during birth and postnatally from breast feeding while 15 to 24-year-olds fall within a young adult category for which unsafe sex is the main risk factor for HIV contraction (4, 5). In both 2016 and 2018, youth aged 13–24 made up 21% of all new HIV diagnoses in the United States (U.S.) (6, 7). Additionally, WHO global health estimates from 2016 show that for females aged 10–14 years, HIV/AIDS was the leading cause of mortality while for their male counterparts, HIV/AIDS was the leading cause of infection-related death and third leading cause of death overall (after road injuries and drowning) (6). Furthermore, for youth living with HIV (YLWHIV); defined as youth under the age of 18 years, AIDS-related illnesses are still among the leading causes of mortality (6). Africa is home to most of the affected children whereby 58% (1.04 million) are from Eastern and Southern Africa and 29% (0.5 million) are from Western and Central Africa (8).

AIDS-related illnesses contributing to mortality among people living with HIV are thought to develop as a result of HIV effects on patients' immune systems (9). Examples of these illnesses are opportunistic coinfections (e.g., tuberculosis, toxoplasmosis, pneumocystis pneumonia) and some cancers (10). Regarding the latter, people living with HIV have a significantly higher risk of developing certain cancers than the general population of the same age (11). HIV infection among cancer patients is also associated with a significantly higher risk of dying from that cancer (11).

HIV-associated cancer risk and survival among YLWHIV has changed over time with the advent of increased access to and uptake of antiretroviral therapy (ART) (8). Prior to ART, cancer risks were reported at >40-fold higher in HIV positive youth (1), with the greatest risks for Kaposi's sarcoma (KS), Non-Hodgkin's lymphoma, and Burkitt's lymphoma (12–15). In the post-ART era, 4 to 14-fold higher pediatric cancer risks have been reported in South Africa (8, 16). It is important to highlight that despite the increased access and effectiveness of ART among people living with HIV, cancer survival among YLWHIV is low in low-resource settings and cancer risk is still higher for children that start ART at an older age and/or who have greater immunosuppression than for those who start ART a younger age and/or who have less immunosuppression (16).

The WHO highlights the importance of early diagnosis for all pediatric cancers and access to treatment for children and adolescents with cancer for better survival (17). Proper access to quality care can result in better prognosis (18). Evidence from high income countries indicates that with proper access to quality care, more than 80% of children with cancer can survive and live full and healthy lives (19–21). This paper provides a narrative review of the factors associated with youth pediatric access to oncological services; and the potential role of economic strengthening can play in improving health outcomes for this population and for all children in low resource settings.

## Methods

This narrative review has the two primary objectives (1) examining access to oncological treatment services among children and (2) discussing the potential role of economic strengthening programs in improving health outcomes for YLWHIV who develop cancers.

### Search Strategy

Articles were obtained through a comprehensive search of MEDLINE, CINAHL, CINAHL Plus, Ovid, PsycINFO, Academic Search Complete, Global Health, SocINDEX, SOCIndex with Full Text, and EBSCOhost's Clinical and Academic Collections using search terms in Table 1. This search was carried out and reviewed for eligibility by two research assistants. These articles were supplemented through Google scholar and references from key articles.

**Table 1:** Search terms and key words used in the literature search.

**Youth terms**	**HIV/AIDS terms**	**Cancer terms**	**Treatment/Treatment delay terms**	**Epidemiology terms**
Pediatric Pediatrics Pediatric* Children Kids Adolescents Youth Kid Adolescent Childhood Young adults—people teens and people in early twenties Teens Youngster Boy Girl Infant	HIV AIDS Human immunodeficiency virus Acquired immunodeficiency syndrome Persons living with HIV Persons living with AIDS People living with HIV People living with AIDS Persons living with HIV/AIDS People living with HIV/AIDS Viral suppression Virally suppressed *HAART *ART *Pediatric ART cART	Cancer Cancers Malignancies Malignant Malignancy Tumor Tumors Neoplasm Neoplasms Carcinoma Carcinomas Oncology Malignant tumors Kaposi sarcoma Non-Hodgkin's lymphoma Burkitt's lymphoma Leiomyosarcoma Cervical cancer Lymphoma HPV-related cancers HIV-related cancers Lymphoproliferative disorder*	Cancer treat* Cancer therap* Chemotherap* Surger* Oncology services radiation adj3 therap* Delay* in Cancer treat* Delay* adj3 cancer diagnosis Delay* in Cancer therap* Delay* adj3 chemotherap* Delay* adj3 Oncology services Abandon* adj3 Cancer treat* Access* adj3 Cancer treat* Access* adj3 Cancer therap* Access* adj3 chemotherap* Access* adj3 Oncology services Abandonment Care Pathways Systems infrastructure	Epidemiology Incidence Cumulative incidence Prevalence Survival analysis Survival rates Kaplan Meier Incidence rates Risk ratio Relative risk Rate ratio Incidence density Prevalence ratio Standardized incidence rate

### Inclusion/Exclusion Criteria

Out of more than 100 articles and internet sources identified from the search conducted over a period of ~4 months, material from 73 sources that provided information related to the papers' objectives was included. These articles include those obtained during the search (43) and supplemented sources (30) obtained from Google scholar searches and reference lists from included articles.

#### Global Burden of Cancers Among Youth Living With HIV

While most YLWHIV live in SSA, cancer studies from this region are scarce. The number of YLWHIV who develop a malignancy in SSA is poorly defined and available data generally focuses on younger children (<16 years). HIV-related cancer surveillance is further hampered by lack of systematic collection of cancer diagnoses in HIV cohorts and absence of HIV status in available local cancer registries.

Of an estimated 429,000 expected new cases of childhood cancer each year, almost 90% will occur in LMICs and over 80% of the global burden of pediatric cancer mortality is in LMIC (22, 23). A review of the literature demonstrated that the burden of cancers and cancer types differs greatly between high income countries and low- to lower-middle-income countries (LMICs) (22, 24) as well as by region (24–26). Additionally, Ward et al. estimated that over 3 million cases of pediatric cancer will go undiagnosed between 2015 and 2030 (22) and 56% of cases in East Africa will be undiagnosed compared to only 3% in North America (22).

Studies from lower income countries in the pre-ART era demonstrated a >40-fold higher risk of developing cancer in HIV-infected youth compared to their general population counterparts, with Kaposi sarcoma (KS), Non-Hodgkin's lymphoma and specifically Burkitt's lymphoma having the greatest relative risks (12–15). While the introduction of and increased access to ART increased survival, studies from South Africa in the post-ART era still find a higher risk (between 14 and 14 times) of developing cancer among YLWHIV (8, 16). Additionally, a retrospective study in South Africa found that HIV-positive children with cancer were generally younger (average age 6 years) compared to HIV-negative patients with cancer (average age 6.5 years) (25, 27). This study also reported higher mortality among HIV-positive children and that the ratio of living children who were cancer-free to the total of children in the respective category, was significantly smaller among HIV-positive participants. Studies from Malawi on Kaposi sarcoma reported that YLWHIV in the post-ART era are still at a high risk for the disease (28, 29).

#### Access to Oncological Treatment Services for Youth

YLWHIV generally have poor survival despite chemotherapy compared to uninfected peers, mainly due to patients' severe immunodeficiency (21, 30). However, survival may be moderated by differences in access to therapeutic options between HIV-positive and HIV-negative children with cancer. Proper access to quality care can result in better prognosis as well as minimal side or late effects associated with disease and treatment, respectively, whereby younger YLWHIV who access care earlier and at lower levels of immunosuppression may have better tolerance and/or responses to necessary high doses of chemotherapy (18, 31). Evidence from high income countries indicates that with proper access to quality care, more than 80% of children with cancer can survive and live full and healthy lives (32). A study in South Africa indicated that YLWHIV receiving antiretroviral therapy (ART) have a substantially lower risk of developing cancer than those who are not on ART [hazard ratio (HR) 0.29] (16). Biggar et al. also found that survival among YLWHIV with cancer was greater for those treated with ART and chemotherapy (33). Yet, numerous children (both HIV-infected and uninfected) in LMICs continue to struggle with access and adherence to care, contributing to childhood cancer deaths in low resource settings globally (17). Hence, timely access to quality healthcare is an important public health concern.

Access to pediatric oncology services is defined in terms of delay, waiting time, as well as treatment adherence and abandonment. Delay is further categorized as patient/parent delay, health care system delay, physician/health system delay, referral delay, diagnosis delay, treatment delay, lag time, and ultimately, total delay (defined as the time from onset of symptoms to initiation of therapy) (18, 34). Total delay in childhood cancer is a major contributor to cancer-related death among children worldwide and many factors contribute to this delay (34, 35). Overall, health system–related factors have been found to contribute more significantly to lag time than parent delays (33, 34, 36).

#### Factors Associated With Delay and Abandonment Among Youth Living in LMICs

**Figure 1:**
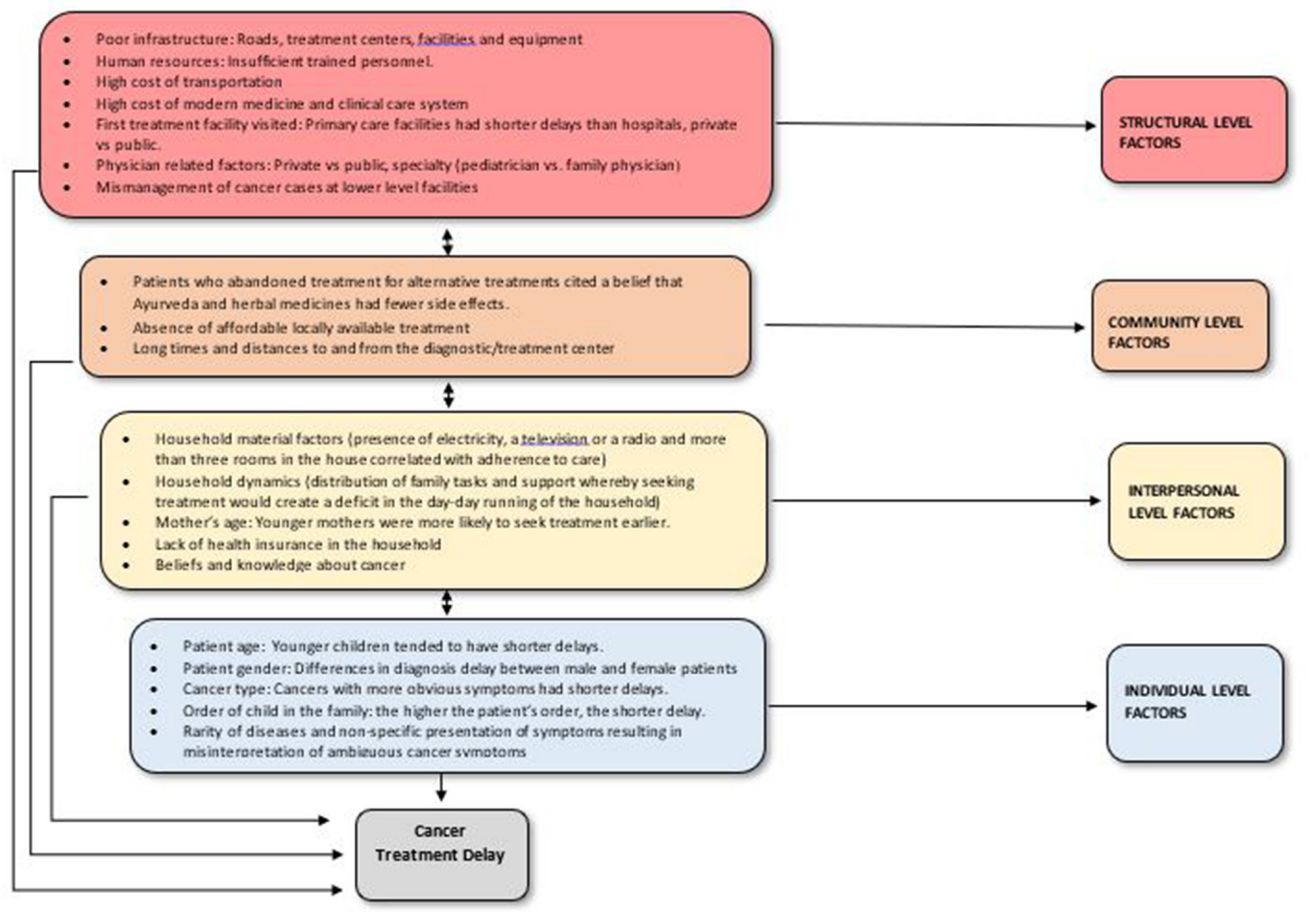
Factors associated with total delay and abandonment of cancer care among children and youth in LMICs.

Several barriers exist to accessing cancer treatment among children and consequently contribute to delay (see Figure 1). As children typically depend on parents/guardians to access health services, individual-level factors are grouped along with interpersonal factors since their influence is ultimately evaluated on an interpersonal basis. Regarding structural factors, most LMICs have only few established cancer centers, whereby cancer services are usually obtained from general surgical or medical departments in non-specialized hospitals (37). These are district or regional hospitals, with little or no information about quantity or quality of the services provided (37). They also typically exist in large urban areas, whereas most of the population lives in rural areas. These hospitals often lack reliable routine pathology, imaging, oncology nursing, and specialized pharmacy departments or have units that offer low quality service and insufficiently trained personnel. This lack or limited scope of pathology services is a major challenge in several SSA countries where there is typically one pathologist for every 500,000 to 1 million people (37).

Lack of affordable local treatments options are amplified by long distances to treatment centers and transportation difficulties (38, 39). Clinical care and transportation costs also play major roles in patient delay. In both Uganda and western Kenya, nearly all guardians rely on public transportation to reach the cancer referral centers, yet transportation costs are high and travel time is long (40). Lack of funds is a critical factor for treatment abandonment (38, 40). Furthermore, the search for an accurate diagnosis and effective treatment was resource-intensive and complicated for many guardians and generally involved multiple visits to different health facilities and care providers (40). Unfortunately, many children remained undiagnosed or were inappropriately treated before getting to cancer referral centers. Studies highlight mismanagement of cancer cases at lower level facilities as a key weakness in health care systems which contributes to late stage of presentation and poor outcomes among children (40). Relatedly, parents who sought alternative sources of care had perceptions of unsatisfactory management by respective health facilities and a lack of confidence in health care systems (39). Alternative treatments were also sought due to the cost of modern medicine and the belief that alternative medicine had fewer side effects (41).

Caregiver education and profession also affect access to cancer treatment services. In a study in Argentina, retinoblastoma patients whose parents had an elementary education or lower had a greater risk of longer patient delay [odds ratio (OR) = 6.34] (18). In Mexico, children whose parents had the lowest level of education had longer delays in diagnosis (OR = 1.4 for fathers and 1.5 for mothers). While there were no significant associations with father's profession, the lag time for mothers with a “blue collar” profession was greater than for both housewives or mothers with academic professions (8.5–13.5 weeks vs. 5.5–6 weeks) (42). Conversely, in Nigeria, children of parents with a higher level of education, who favor private hospitals over public ones, suffered longer diagnosis delays despite being more economically empowered (43).

In a study conducted in Latin America, Asia and the Caribbean, household assets correlated with adherence to care (41). In Uganda, household dynamics (e.g., distribution of family tasks, guardian's role as caretaker, and social support) contributed to patient/parent delay whereby seeking treatment created a deficit in the day-to-day running of households (40). Consequently, parents without a support system for other children in the home and/or someone to take over their tasks had longer delay. Parent delay among Ugandan guardians was also influenced by guardians' beliefs about the curability of cancer, health system delay, and by guardians' perceptions of cancer as a contagious disease (41). Hence, understanding the role of cultural/community context when describing barriers and designing interventions is essential.

A study in Israel, a high-income country, also observed a significant positive correlation between child's age at diagnosis and lag time whereby the lag time increased as the child's age increased (44). Diagnosis delay was shortest for children between the ages of 0–2 years despite no significant differences in histopathology, grade or location of tumors, and parental persistence (number of consultations before diagnosis) across age groups. The association with child's age was explained by the expectation that younger children have closer parental supervision, which may facilitate the recognition of symptoms and signs, as opposed to older children and adolescents who more often initiate the recognition of signs and symptoms themselves. Adolescents may also be more likely to undervalue symptoms and delay calling attention to them, resulting in increased delays. Moreover, if the child was the oldest, the parent's delay was shorter, underlying birth order as a critical factor. Patients with younger mothers were more likely to seek treatment earlier vs. those with older mothers. This was attributed to younger mothers having less experience in taking care of children and therefore being more likely to seek medical attention earlier (39).

Cancer-related factors such as the cancer type, symptoms, stage, and tumor site/location also contribute to delays. Cancer type was significantly associated with delayed time to diagnosis especially when comparing different groups of cancers to leukemia, an effect which persisted even after adjusting for age, sex, and race (40). The timely diagnosis of cancer in children was found to be further complicated by the rarity of the disease and the non-specific presentation of symptoms as well as misinterpretation of ambiguous cancer symptoms by patients, parents, and physicians (18). Patients with abnormal masses had shorter diagnosis delays, patients with rare symptoms had shorter parent delay, fast-growing tumors had shorter delays than slow-growing ones and patients who presented with pain as the primary symptom had significantly longer total delay and physician delay (35, 38). Patients with disseminated disease also had significantly longer delays compared to patients with localized disease (45).

The type of physician seen first (private vs. public), physician specialty (pediatrician vs. family physician), type of facility (private vs. public), health insurance, and the fear of visiting tertiary health care institutions also contributed to delays among children with suspected cancers (43). When the first health consultant was a private physician, the lag time was shorter than when it was a physician at a public clinic; and both lag time and physician delay were shorter among children seen by pediatricians compared to those seen by family physicians or other specialists (44). Finally, a lack of health insurance combined with the initial use of alternative medicine was also significantly associated with longer patient delays (36).

This review could not identify studies that compared access to oncological services between HIV-positive and negative children. Yet, two U.S.-based studies focused on adults demonstrated that people living with HIV were less likely to receive cancer treatment and that the delay between diagnosis to treatment was also significantly longer than that of HIV negative individuals (41). The lack of resources and varying treatment availability within and across different regions makes the evaluation of cancer in the pediatric HIV population particularly challenging. The weaknesses in health care infrastructure for diagnosing cancers along with limited epidemiological expertise in LMICs, especially in SSA also result in sparse data on the burden of AIDS defining malignancies and other associated cancers (45).

#### Current Interventions and the Potential Role of Socio-Economic Empowerment in Improving Access

Children with cancer in LMICs not only experience multiple barriers to treatment access, but they also have lower childhood cancer survival rates (15, 46). The reasons for the low survival rates are multiple, interrelated, and associated with factors that affect access to treatment services, treatment adherence as well as abandonment. The small number of facilities providing cancer treatment and supportive services along with lack of well-trained multidisciplinary staff are further aggravated by poor nutritional status of children and late patient presentation to the health centers and/or specialized personnel (47). Hence, further development and evaluation of interventions aimed at improving access to oncological services for youth is required.

Despite improvement in outcomes for pediatric cancer patients in LMICs, many challenges persist. Interventions including targeted health education of guardians on the importance of early care for children's illnesses as well as advocacy and education of health workers to facilitate the identification of early warning signs of childhood cancer and foster timely referral and diagnosis are crucial for patient survival (40). Parent support groups also offer vital support in providing medications, housing and psychosocial support (48). Additionally, the establishment of cancer centers and “twinning” partnerships with institutions from high-income countries in many LMICs, especially in SSA countries, have led to infrastructure improvement as well as personnel and facility-capability to provide proper cancer treatment and care (47, 48). Twinning partnerships occur across multiple levels including the local public sector, private-sector and international organizations and include education, care-linkage, mentorship (e.g., regular online conferences in international partnerships), and in some cases, funding (47).

Similarly, regional collaborations between cancer centers have driven progress in pediatric access to treatment and survival (49). Developing national protocols and guidelines for treatment that account for local contexts (e.g., unavailability of certain drugs or treatment modalities) is also another important step (47). For instance, the committee for Pediatric Oncology in Developing Countries published treatment guidelines for children with common and curable cancers (including YLWHIV) (49, 50). Other considerations include improving supportive care services, infection control programs (especially opportunistic infections among YLWHIV) and nutritional support benefits (47).

Developing sustainable and contextually relevant interventions, especially in resource-poor settings is imperative. While current interventions are vital and have resulted in increased access and government involvement in oncological care, socio-economic factors, and treatment-related costs play a significant role in the cancer-care pathway (40). Hence, interventions increasing the ability of families with young cancer patients to engage with the healthcare system may lead to better health outcomes.

This economic empowerment can be achieved using an asset-based model, which is an integrated approach for building human, social, and economic capital (51, 52). The asset theory posits that asset building (e.g., savings, educational opportunities, and economic opportunities in the form of small businesses or income generating activities) has important long-term positive benefits (53, 54). Asset theorists have described these as “asset effects” (55) which include acquiring a sense of economic security, self-confidence, hope, and responsibility (56). This empowerment could facilitate families' access to formal financial products (e.g., savings and credit lines) to meet the healthcare needs such as treatment for HIV and cancer, foster adherence and consequently improve health outcomes (57).

People living in poverty acquire personal financial resources either through savings or loans (58). However, if the latter are not managed well, they can increase the financial burden on recipients rather than facilitate improved quality of life (59). Therefore, saving programs would be the better option to address disparities in treatment access and help families with children suffering from cancer.

Asset-based development through savings interventions has been used with success to improve quality of life. For example, the Suubi projects (Hope in Luganda, the local language in the region) in the Greater Masaka region in Uganda have employed an asset development approach in working with orphaned and vulnerable children in poverty -including YLWHIV, which among other components, involve setting up a Child Savings Account (CSA) (58, 59). CSA is a matched savings account held in the child's name in an officially registered financial institution or bank (60) and for youth and their families to use for school-related expenses, post-primary education, and in the case of YLWHIV for medical expenses, or to start small family business development projects (53, 60). Tested using randomized controlled trials, results indicate that this program is effective in reducing children's economic barriers to attend school, supporting the argument that such programs can address family-level and child poverty, which could in turn improve health outcomes. More specifically for YLWHIV, in a large sample of Ugandan adolescents between the ages of 10 and 16 years and living with HIV, the intervention was found to significantly improve viral suppression, the primary marker of ART adherence (61, 62). It is possible that the promotion of financial stability may have addressed transport costs and food security, both of which are factors identified as barriers to ART adherence (63–66). Given these results, economic empowerment interventions may also effectively address barriers to cancer treatment and care.

#### Next Steps

Capacity building activities across all levels of health care systems are key to the care and treatment of children with cancer in LMICs—both with and without HIV infection. This approach requires collaborative work based on local stakeholder and government buy-in in order to set the priorities because they know best what they need and is feasible. Strengthening and expanding comprehensive pediatric cancer registries, especially in SSA will provide opportunities for research and innovative treatment approaches to improve survival. Collaboration between new and existing cancer and HIV registries for children and youth can further facilitate this process. Larger longitudinal studies are also needed to determine incidence of cancers in HIV-positive and HIV-negative children to better characterize the association between seropositivity and cancer.

Established HIV-care systems and mechanisms at both local and regional levels should be utilized to provide cancer diagnosis and linkage to treatment services. Setting up centralized cancer treatment centers, partnering with established cancer units, establishing locally adapted protocols, and getting financial support from the government are all helpful in reducing treatment abandonment (41). Careful consultation is also required between the different subspecialties involved in the care of these children, including the multidisciplinary HIV team, infectious disease, and oncology subspecialists, so that decisions about cancer treatment are made in the context of ART, and consider the treatment required for other HIV-associated disorders and patient quality of life. As cancer becomes more frequent in both people living with HIV and YLWHIV, physicians need to better understand and agree on when to treat, when to palliate, and, with treatment, how to minimize complications while achieving remission.

Research on diagnosis delays in childhood cancer is also important. There is also a need for more studies to investigate the potential impact of delays on prognosis outcomes. Future studies should study access to oncological services among YLWHIV, a high-risk group for pediatric cancer. Examining the impact of patient and provider/healthcare-system delays on disease severity and prognosis as well as the factors influencing these delays would inform effective policies and programs aimed at eliminating obstacles in the cancer-care pathway for youth with cancer.

Finally, better strategies to reduce treatment abandonment and improve survival in childhood cancer in the developing world are still needed. Increasing awareness of childhood cancers and addressing structural and socio-economic factors impacting access to cancer treatment services can be considered as potential strategies. Moreover, complementing current structural interventions with the adoption of asset-based models to improve access and adherence to oncological treatment could be vital to pediatric cancer patients, especially YLWHIV.

## Conclusion

Pediatric cancers among youth are rare but serious life-threatening conditions. YLWHIV are particularly at a higher risk of developing and dying from cancer than their general population counterparts. Pediatric cancer patients have limited access to oncological services and health-system-related factors play a major role in delayed access to treatment. Failure to meet treatment costs is a major contributor to treatment abandonment. This paper discussed the potential role of socio-economic strengthening and provided evidence that asset-building savings programs can increase financial security and subsequently result in better health outcomes. Further exploration, development, and evaluation of interventions to address gaps and challenges to access to oncological services among YLWHIV and all children are crucial next steps.

## Author Contributions

FS, OS, and KJ were principal investigators for the NIH funding. RK drafted the manuscript. OS, FS, and KJ reviewed and made substantive edits to the manuscript. All authors contributed to the article and approved the submitted version.

## Conflict of Interest

The authors declare that the research was conducted in the absence of any commercial or financial relationships that could be construed as a potential conflict of interest.

## Figures and Tables

**Table:** 

**Abbreviation/Acronym**	**Definition**
YLWHIV	Youth living with HIV/AIDS. Youth are defined as individuals between the ages of 0 and 24 years. Therefore, this definition includes younger children 12 years and under, teenagers and young adults (18–24)
LMICs	Low- and middle-income countries as defined by the World Bank
LIC	Low-income countries as defined by the World Bank
WHO	World Health Organization
ART	Antiretroviral therapy
SSA	Sub-Saharan Africa
HR	Hazard ratio
CSA	Child Savings Account
